# Research Data Management and Libraries: Relationships, Activities, Drivers and Influences

**DOI:** 10.1371/journal.pone.0114734

**Published:** 2014-12-08

**Authors:** Stephen Pinfield, Andrew M. Cox, Jen Smith

**Affiliations:** Information School, University of Sheffield, Sheffield, United Kingdom; World Health Organization, Switzerland

## Abstract

The management of research data is now a major challenge for research organisations. Vast quantities of born-digital data are being produced in a wide variety of forms at a rapid rate in universities. This paper analyses the contribution of academic libraries to research data management (RDM) in the wider institutional context. In particular it: examines the roles and relationships involved in RDM, identifies the main components of an RDM programme, evaluates the major drivers for RDM activities, and analyses the key factors influencing the shape of RDM developments. The study is written from the perspective of library professionals, analysing data from 26 semi-structured interviews of library staff from different UK institutions. This is an early qualitative contribution to the topic complementing existing quantitative and case study approaches. Results show that although libraries are playing a significant role in RDM, there is uncertainty and variation in the relationship with other stakeholders such as IT services and research support offices. Current emphases in RDM programmes are on developments of policies and guidelines, with some early work on technology infrastructures and support services. Drivers for developments include storage, security, quality, compliance, preservation, and sharing with libraries associated most closely with the last three. The paper also highlights a ‘jurisdictional’ driver in which libraries are claiming a role in this space. A wide range of factors, including governance, resourcing and skills, are identified as influencing ongoing developments. From the analysis, a model is constructed designed to capture the main aspects of an institutional RDM programme. This model helps to clarify the different issues involved in RDM, identifying layers of activity, multiple stakeholders and drivers, and a large number of factors influencing the implementation of any initiative. Institutions may usefully benchmark their activities against the data and model in order to inform ongoing RDM activity.

## Introduction

Data management is now a major challenge for research organisations. Vast quantities of born-digital research data are now being produced in a wide variety of forms and at a rapid rate in universities, creating the so-called “volume”, “variety” and “velocity” challenges of data [1], [2]. This “data deluge” generates a need to develop policies, infrastructures and services in institutions to manage data, with the aim of assisting researchers in creating, collecting, manipulating, analysing, transporting, storing and preserving datasets. In certain circumstances, there may also be a need to share data either amongst specific groups or openly. Despite its challenges, there is growing recognition that sharing data widely can create benefits, including allowing the verification of research outcomes, enabling contribution to wider data gathering scientific activities, and facilitating the reuse of data by others for subsequent research [3], [4]. Research funders, conscious of the need to encourage scientific good practice and to achieve greater value for the research they sponsor, widely encourage – indeed, increasingly require – particular standards of data management and sharing to be followed [5], [6]. Such requirements serve to emphasise the need for effective research data management within institutions.

As different approaches to research data management (RDM) are developing in universities and other research organisations, different stakeholders have become involved, including support services staff as well as Faculty themselves. University libraries have moved into this space and are increasingly seen as major contributors to RDM activity in general and in the design of research data services (RDS) in particular [7]–[14]. The study presented here makes a contribution to the ongoing discussion in the research and professional literature by reporting results of qualitative research consisting of interviews of library professionals in UK universities discussing their involvement in institutional RDM activity. It is designed to provide an in-depth insight into the thinking of library staff involved in RDM as they confront the wide range of challenges involved.

The paper begins with a brief overview of the research context, discussing in particular the concept of RDM and the literature on librarians' roles in it. It then goes on to outline the objectives of the research undertaken and the research methodology used. The results then presented provide perspectives on the relationship between the library and other institutional stakeholders involved in RDM, the sorts of RDM activities being carried out, the drivers for those activities, and the main factors that influence their shape. From the data presented, a model is constructed which attempts to capture the main aspects of an RDM programme in an institution.

## Research Context

Research data management is defined by Whyte and Tedds as, “the organisation of data, from its entry to the research cycle through to the dissemination and archiving of valuable results” [15]. As Cox and Pinfield observe, RDM “consists of a number of different activities and processes associated with the data lifecycle, involving the design and creation of data, storage, security, preservation, retrieval, sharing, and reuse, all taking into account technical capabilities, ethical considerations, legal issues and governance frameworks” [8]. Such activities and processes are needed for a wide variety of different forms of data ranging from large-scale calculations derived from high-performance computing facilities, through results of scientific experiments, to audio recordings of interviews. RDM is therefore a highly complex set of activities involving an array of technical challenges as well as a large number of cultural, managerial, legal and policy issues.

The library's involvement in RDM has been discussed in the literature particularly in the last 5 to 6 years. Early contributions, such as those by Delserone [16], Henty [17] and Lewis [7], set out the case for library involvement. Later works, such as Corrall [18] and Cox, Verbaan and Sen [19] further discuss the range of possible roles. Lyon [20] identifies a number of opportunities for libraries but also major challenges in developing the capacity and capabilities to carry out RDM. Pryor [12] summarises these opportunities and challenges by talking about “the re-purposed librarian”. Procter, Halfpenny and Voss [21] place the role of the library in a wider institutional context, emphasising the need for libraries to work in partnership with IT services and academic staff.

Further studies have attempted to gather empirical evidence of the extent of actual library involvement in RDM. Tenopir and colleagues [10], [22], [23] report the results of a large-scale study of US libraries' involvement in research data services design and implementation, showing activity in technical infrastructure development as well as support and advisory services. Corrall, Kennan and Afzal [9] report results from a survey of university libraries in Australia, New Zealand, Ireland and the UK carried out in the first quarter of 2012, identifying widespread early activity albeit constrained by knowledge and skills gaps. They assess early work in these countries, particularly the UK, to be somewhat behind those of the US. Cox and Pinfeld's study [8] of UK libraries presents survey results from late 2012 showing progress in the UK, with policy development involving the library in particular proceeding rapidly and a high strategic priority being given to some RDM activities especially around training and advisory services.

Empirical studies such as these have, however, to date largely been quantitative in their approach. Whilst they have identified a range of important developments, they have perhaps also highlighted the need to gain a richer and more nuanced picture of ongoing developments. The current paper goes some way to achieving that by making an early qualitative contribution, to date an approach largely absent from the literature. It complements work recently published by the authors based on the same dataset focused specifically on analysing the challenges of RDM within the framework of the ‘Wicked Problem’ concept (“unique, complex problems which are defined differently by different stakeholders making them particularly intractable”) [24]. The current study also follows on from quantitative work by Cox and Pinfield [8], allowing issues identified there to be explored in more depth.

Complementing empirical studies, a number of researchers have generated models of various aspects of Research Data Management. For example, Jones, Pryor and Whyte delineate a “Components of RDM support services” model [25], organised within a simple research life cycle framework (further discussed by Jones [26]). There is an overarching “Policy, strategy and business case”. Then there are a number of components relating to different stages of research, namely: support for data management planning; managing active data; data selection and handover; sharing and preserving data (including data repositories and catalogues). “Guidance, training and support” covers support services to researchers throughout the process. The model gives detail about the types of services that could be offered around RDM, and one of its strengths is its link to a simple data lifecycle concept. It could be seen as a successor to the well-known Digital Curation Centre (DCC) Lifecycle model, which is rather complex and focuses more on data curation than on researcher support. Complementing the Service component model, Whyte proposes a process by which services might be brought into being, through a six-step pathway of envisioning, initiating, discovering, designing, implementing and evaluating [27]. The author sets out the tools that have been developed by the community to support each step of the process. He acknowledges some of the challenges, particularly in the diversity of research communities, but does not provide a detailed consideration of any constraints on the service delivery side. Mayernik et al provide an outline of a “Data Conservancy” model of RDS based on work at Johns Hopkins University in particular. This covers both technical components (such as software and infrastructure) and organisational components (such as policies and funding strategies) [28].

Good practice has also begun to emerge as institutions have set out to develop RDS and have published lessons learned based on real-world experience. Experiences in institutions such as Edinburgh [29] and Oxford [30] in the UK, and Purdue [31] and Johns Hopkins [32] in the US, have highlighted some important issues at institutional level. Differences in national approaches between Australia and US have also been usefully explored [33]. However, even the advanced institutions identified in these publications offer only hints of what a fully developed RDS may look like. At the time the interviews reported in this paper were carried out, many institutions were clearly only taking their first steps towards finding a pathway.

The research had four main objectives designed to analyse the library's contribution to RDM within the wider institutional context:

1. To examine roles and relationships involved in RDM
2. To identify the main components of RDM programmes
3. To evaluate the main drivers for RDM activities
4. To analyse the key factors influencing the shape of RDM developments

In this way, the research was designed to yield a rich understanding of current institution-wide RDM developments as contributed to and perceived by one of the major stakeholder groups – the library.

## Methodology

The data presented here derives from 26 semi-structured interviews of library practitioners from different institutions in the UK. These interviews were the second phase of a project which began with an online survey of UK academic libraries reported by Cox and Pinfield [8]. Respondents to the survey were asked to volunteer to take part in detailed follow-up interviews as part of their responses, and therefore interviewees represented a self-selected group of practitioners, probably engaged with RDM to a greater degree than the community as a whole (although a number of participants were only just beginning to address RDM issues when interviewed). Participants came from a number of different sorts of institutions (research-led and teaching-led institutions, large universities and small institutes) and were either senior library managers with a strategic overview or middle-level managers with direct responsibility for RDM.

The approach adopted for the research was given approval under the University of Sheffield Information School ethics approval process as overseen by the University of Sheffield Research Ethics Committee. The University Research Ethics Committee monitors the application and delivery of the University's Ethics Review Procedure across the University. The process as carried out was based on the principles of voluntary contribution, informed consent and anonymised reporting. Participants, who had volunteered to take part by providing their contact details in the previous online survey reported by Cox and Pinfield [8], were sent Information and Consent documents in advance of the interviews which explained details of the research approach and their part in it. Participants were given the opportunity to ask questions about these via email before the interviews were scheduled. At the beginning of the formal telephone interviews, each participant was given the opportunity to ask questions verbally regarding their participation and asked to confirm their consent. Verbal consent was deemed appropriate as interviews took the form of pre-arranged telephone conversations with self-selecting volunteer participants. 25 of the interviews were conducted by telephone, with one face-to-face, at the request of the particular participant.

The interviews covered three main areas: firstly, the current state of RDM activity in the institution; secondly, the skills needed to carry out RDM services; thirdly, the story of how RDM policy and services have developed in the institution. Interviewees were encouraged to talk in detail about their experience of the issues involved, including the library's role in delivering RDM and in developing RDM-related strategies and policies, with interviews lasting 40 minutes on average. Following piloting, interviews took place between March and June 2013. Interviews were recorded and then fully transcribed for analysis.

The interview transcripts were analysed in NVivo following a thematic analysis approach [34]. All three authors initially familiarised themselves with the data with a thorough reading of the transcripts, involving memo writing. Initial codes were then identified based on key features identified in the data, including “semantic content or latent” features [34]. Codes were applied to the transcripts in NVivo, and reviewed and refined on a number of occasions by the authors following review. After initial coding of all of the transcripts, major themes were identified from the data, with code structures refined and thematic maps developed to reflect these. Codes and themes were reviewed and again refined upon further analysis of the data. The data was then used to construct a model of an RDM programme in the institution from a library perspective which was designed to capture the key themes highlighted in the interviews (presented below).

## Results

### The library and institutional RDM activities

All participants reported that the library was involved in institutional RDM activities and expressed the view that the library should be. There were, however, significant reported differences in the roles currently played by libraries and how these related to other stakeholders. There were also differences in views on how these roles and relationships should be developed. Some argued strongly for the library taking a leadership role:

*“I think there are a number of areas where the library does have a distinctive role to play here. I certainly don't think libraries should be shrinking away from this at all. Lots of opportunities for leadership.”*

Areas highlighted where the library could lead focused on the formulation of policy and creation of guidance documentation (although this may reflect the fact that for many those areas of development were currently priorities). Some participants reported that the library had been tasked with leading the development of a policy in response to the new requirements of the UK's EPSRC (Engineering and Physical Sciences Research Council) [5]. One respondent reported that it was likely that the library would take a leadership role partly because of the reluctance of other support services to do so:

*“…the feeling is that other than the library, most service areas aren't really going to want to, to tackle it, take a lead on it, and so talking to IT staff in the library, there was a feeling that this is very much a library area…”*

Other interviewees reported an involvement with RDM but in a contributory rather than a leadership role:

*“…we have contributed to [policy development] and commented on a document and attended meetings and assisted…[the] research [office] who have been leading on developing the policy.”*

Some participants saw the library as being in an ambiguous position and were themselves uncertain about its role in RDM. Current work was seen as a way of helping to resolve that uncertainty:

*“This is one we are trying to figure out ourselves here, work out exactly what the library's role is.”*

There was sometimes disagreement even within individual library organisations about whether in both the short and long-term the library should lead on RDM issues. One participant highlighted some of the issues:

*“At the most senior level, the library needs to take a strategic decision how active they want to be in pitching for data management because it's there for the taking if they want it, in many institutions. Or that it gets kicked in their direction because there is no other obvious place for it to go, but what I would say from the experience here, it has to be a cross-institution service. Now somebody needs to own it, and I don't know that the library is the right place to own the whole shebang. I think the library has strengths that it will bring to certain areas of the service and therefore absolutely should be involved in policy and can be a driving force behind the policy, which I think in [this institution] it has been, in conjunction with again our colleagues in IT who we work closely with.”*

In some cases, participants observed that various aspects of RDM were being led by different players in the institution. However, the extent to which this was planned and explicitly agreed, and the extent to which it was emergent and implicitly accepted, varied. However, all interviewees agreed that a collaborative approach to addressing challenges within the institution was essential.

*“Nobody can own this on their own. They are going to have to really appreciate what those other partners' roles might be and where their expertise is and where that expertise needs to be drawn in and listened to.”*

The participants provided accounts of ongoing collaborative work involving most commonly research support services and IT services. Other support departments mentioned included legal advisory services and records management services as well as senior academic staff. Whilst participants reported some differences in perspectives with these groups and also some disagreements, ongoing collaborative working was taken for granted. Work was often overseen by an academic member of the institutional executive board, typically the Pro-Vice-Chancellor or Vice President with responsibility for research (or equivalent), and may also have involved other senior academic staff.

In some cases, the library was reported to have taken up a coordinating role between the various support services staff and Faculty involved. One respondent reported that the library was in a good position to take an ‘honest broker’ role:

*“I think we can be quite good at just bringing different people together… Although [the library is] a central service, I think it's often perceived as being quite a neutral safe place… So you can actually bring together…[and] you can facilitate those discussions in a way which isn't very threatening… I think also we have quite good relationships with our researchers, and we understand the nature of their research perhaps a bit better, we come at it from a different angle, I think perhaps than IT [services] might do.”*

The picture of how libraries are involved in RDM therefore shows considerable variability and at the time of the interviews was still in flux. RDM was seen as a professional concern, but the degree of involvement of the library was uncertain. This perspective was, of course, only a partial picture of the relationships within institutional RDM activity. Other stakeholders who also have a vital role in defining approaches, did not contribute directly to this research project and therefore perhaps need to be covered in future research to create a rounded picture.

### Components of an institutional RDM programme and the library

Emerging from analysis of the narratives of RDM developments provided by participants, a number of key components of an institutional RDM programme can be identified. The term ‘programme’ is used here to describe the various activities, such as policy development and technology implementation, that together constitute concerted effort in a particular area (similar to the use by Kennan [35] in relation to an institutional repository “program”). The programme components are:

- **Strategies:** defining the overarching vision for research data management within the institution and how it relates to the institutional mission and priorities, and outlining major developmental goals and principles which inform activity.
- **Policies:** specifying how the strategies are to be operationalised through regular procedures, including not just an RDM policy but also a set of complementary policy frameworks covering issues such as intellectual property rights and openness that may be relevant.
- **Guidelines:** providing detail on how the policies will be implemented often written from the point of view of a particular user group (such as those within a particular disciplinary area) and defining specific activities, and roles and responsibilities.
- **Processes:** specifying and regulating activities within the research data life-cycle including research data management planning for individual projects, data processing, ingesting data into central systems, selecting data for preservation, etc, and involving the use of standards and standardised procedures wherever possible.
- **Technologies:** underpinning processes with technical implementations including data repositories and networking infrastructures allowing for storage and transport of data.
- **Services:** enabling end-user access to systems and providing support for research data life-cycle activities (including supporting the creation of data management plans, providing skills training, and delivering helpdesk services).

Of these, much current activity reported by participants clearly focused on the development of an institutional RDM policy. Policy development was typically sponsored in institutions by the Pro-Vice-Chancellor for research and overseen by the institutional research committee. In many cases, a working group or task force had been set up, often chaired by the Pro-Vice-Chancellor (or nominee), and consisting of senior representatives from support services (including the library, IT services and research support services) and the academic community. Commonly, this group had a formal reporting line to the research committee, with ultimate responsibility for approval of the policy with that committee or sometimes with the university executive board or senate.

Policies normally took the form of a set of high-level principles. In some cases, they were developed relatively quickly, often drawing on existing good practice. One participant described the process of, “looking at other people's policies and pulling the bits that we think are relevant to us”. Policies were often explicitly seen as working documents, developed iteratively and requiring ongoing revisions as a result of consultation:

*“We…went through some drafts and iterated principles and gaps and concerns that people had, in the policy until we reached a version that whilst we call final because it's gone through Senate, we have always said we consider it to be a policy that will go back to Senate. We will take a year or two's worth of feedback on the policy and then probably another version might go back with some change…as we go through trying to work out how we best support that policy.”*

Policy development was often explicitly seen as informing a wider set of developments within the institution:

*“You want some framework, so you do need a policy, and a road map, and a sense of where you are going. You do need that to inform longer term strategic investment, particularly in key areas like storage, equipment, and staffing for services. And you need something which outlines roles and responsibilities in terms of research data management, and this has to in some way be endorsed from the top.”*

There was, however, widespread agreement amongst participants that this ‘top-down’ approach also needed to be combined with ‘bottom-up’ engagement:

*“I think you need some leadership from the top and they can set things running, but unless you get people further down interested and engaged it's not going to work, just telling people to do it never seems to work in universities.”*

A number of participants described how the development of a policy was also associated with some kind of audit or consultation process with the wider academic community to gauge views, identify current activities and understand expected requirements with regard to RDM. In some cases these exercises were somewhat informal, consisting, for example, of a set of meetings with faculty representatives:

*“At the moment like many other places we are doing a bit of requirement checking, so we have got a series of interviews and meetings with different departments looking at individual cases etc.”*

In other cases, the institution had undertaken a more structured data audit process. In a small number of cases, participants reported carrying out a formal Data Audit Framework (DAF) exercise:

*“…there is a project which is actually currently doing a requirement analysis, using a version of the DAF tool, to try and find out more about people's activities…we are trying to build an institutional picture, to develop a project with multiple strands that will look at things like storage, long-term archiving, preservation, training, support for data management planning, etc.”*

However, such a formal process was not the norm, and in many cases, the level of engagement with a *broad* range of academic staff either in policy development or other activities was up to then acknowledged to be limited. Some participants commented on low levels of interest from academic staff with policy development despite attempts to widen discussion through, for example, a survey:

*“Nobody in the survey commented on the policy, and nobody has been e-mailing in commenting on the policy, and as I say when it went to the faculty research and innovation committees, it went to every…one …before it went to Senate, and the changes were really minor, so I think because it was such a high-level document, people were just shrugging and like “Yes, whatever”. Because it doesn't actually spell out in black and white terms what you have to do in practice, and there is no notion of any consequence if you don't do it.”*

In many cases, the involvement of academic staff was often now seen as a priority particularly associated with the production of guidelines to support the implementation of the policy – something that was seen as an emerging priority for those institutions that had already developed a policy at least in draft form:

*“So then there is all the policy stuff which is pending just now, because we did some drafts in May that were very light touch, and when we have finished our machinations that we are doing just now and asking people, we will do an update in the summer. And then we will update it again next summer…and then we will have some…clearer process guidelines…”*

Guidelines or procedures documentation was being designed to spell out more clearly the roles and responsibilities of different groups in the institution and describe processes to be undertaken as part of RDM by them. Often the need for further guidance was mentioned by participants in relation to specific issues, such as intellectual property and copyright, or metadata production. Producing guidance of this sort was seen by a number of participants as the next natural step following the development of a high-level policy.

Significantly, many of the activities reported to be taking place in institutions were concentrated at either policy or procedural level. Although its importance was acknowledged, there was little discussion amongst participants about overarching strategy for the institution and its link with the management of research data. Whilst some policy documents may explicitly map RDM to existing institutional strategy, engagement at this level was limited. This is somewhat surprising giving the formal involvement of senior university governance groups in policy development and seems to be indicative of RDM being regarded as a procedural rather than strategic issue in many institutions.

Current activities sometimes also involved use of small-scale projects to explore issues and test approaches. A willingness to experiment was seen as important by many participants, with one highlighting:

*“Flexibility and a willingness to learn new things, and to learn through trial and error.”*

In a number of cases, these projects were explicitly seen as pilots of potential future services. One participant described in detail the thinking behind such an approach to ‘pathfinder’ activities, combined with policy creation, in terms of future service development:

*“…we are starting from a relatively low base, and we are in the process of building an integrated, coherent research data management service. This builds on the work of a research data management working group that was set up approximately 2 years ago…the group was set up to respond to the requirements from funding councils for institutions to have a road map for research data management and was looking at how that could be achieved within [the institution]. The recommendation from that group was for a pilot project to test what a research data management service would look like. Bringing together the different… support services across the institution which are already involved in research data. The working group also proposed a draft research data management policy, so at the moment we are just about to start that pilot project, to set up a research data management service to work with a few selected researchers from across the institution to see how that process will work in practice. So we are at an early stage, but the project is really to bring together the existing activities across the institution and to develop them into a more coherent service.”*

However well-developed such plans were, participants nevertheless often adopted conservative views of what could realistically be achieved in the short term, partly to manage expectations, partly out of realism.

Participants acknowledged that RDM required significant levels of technology infrastructure development. Library involvement in this often focused on the institutional repository. However, there was uncertainty about the extent to which the library's involvement in repository development was relevant for RDM activities. Some participants were sceptical as to whether the repository services run by the library could be scaled up to accommodate large datasets. Very often, therefore, data storage and other technical infrastructure was identified as outside the library remit, with IT services identified as leading in this area. Similarly, few participants mentioned the issue of the development of standardised processes and workflows making use of the technical infrastructure. Key issues associated with managing the data life-cycle including setting up processes associated with ingest of data, developing standards for creation of metadata, producing protocols for selecting data for preservation, whilst acknowledged to be important, tended to be beyond the current thinking of most participants.

Apart from provision of systems, major services mentioned by participants focused on training and advisory services, most of them in the early stages of development. One interviewee described the delivery of a formalised data management plan advisory service which was now in operation in the institution. Other services discussed, but not widely delivered, included a data catalogue, and data discovery services (for both internal and external data). These were seen as areas of potential library involvement.

Thinking in this area and others often extended beyond the institution. All participants were aware of good practice elsewhere, either in the UK or internationally. Many were aware of developing international standards and services. In some cases they actively collaborated with others outside their own institution in projects or through sharing experiences in pre-existing interest groups such as Research Libraries UK. They also valued the role played by organisations such as Jisc and the DCC in highlighting good practice and providing training.

In general, there was, however, a tone of provisionality running through the comments of many of the participants in this research. This was highlighted in the way a significant number of interviewees contextualised their responses with the proviso that it was “early days”. This was a commonly repeated sentiment in the interviews and characterised a general uncertainty around the specifics of the library's role in RDM, even if the principle of the library being involved was widely accepted. The participant described their activity as “feeling our way” seemed to be summarising the views of many. The emphasis on development of an institutional policy and project work was seen as a way of establishing a platform from which other components of RDM could develop. These other components, including the creation of detailed guidelines, processes, technologies and services were seen as important, but were often still nascent. Guidelines development and early pilot services were, however, beginning to emerge as significant areas of activity.

### Drivers for institutional RDM and the library

A number of drivers for RDM developments were identified in the interviews as being important at an institutional level:

1. **Storage:** the need to provide immediate storage facilities for a wide variety of datasets at a scale which anticipates the future requirements of researchers and in a way that represents value for money and is convenient to use.
2. **Security:** the requirement to ensure that data, particularly that which is confidential or sensitive, should be held securely with relevant authentication and authorisation mechanisms in place.
3. **Preservation:** the need for medium and long-term archiving of data with associated selection protocols and preservation activities along with a supporting technical infrastructure.
4. **Compliance:** the need to comply with the requirements and policies of other relevant agencies, particularly funders, as well as legal obligations, such as data protection, and industry good practice.
5. **Quality:** the imperative to maintain and enhance the quality of research activity in general in order to demonstrate the robustness of findings and enable results verification and reproducibility (partly derived from but not limited to the quality of research data itself).
6. **Sharing:** the need to share data amongst targeted users and also to provide mechanisms and systems to enable open access to data where appropriate.
7. **Jurisdiction:** the development of a professional narrative around the need to be involved in RDM and how this impacts upon other stakeholders in the institution.

Drivers 1 to 6 were identified by the research team from previous work and confirmed by participants in their responses. Driver 7 emerged from the interviews, being implicit in many of the comments of participants (although the word ‘jurisdiction’ itself was not used). Participants made some interesting comments on the role of the library in particular and the institution in general responding to these drivers.

Participants agreed that the storage of data was a very high institutional priority and a major driver for RDM activity:

*“I think storage is a big issue. So in terms of difficulty of solving this in an affordable way, I would put storage as number 1…I think the storage comes first in terms of you solve that and some of the other things are…part of that.”*

Concerns were expressed about researchers developing local storage solutions for their data, ranging from the use of small-scale portable storage devices to large-scale server-based installations. Such local solutions were seen to create risks associated with resilience and security in particular. Some participants reported that such developments may reflect dissatisfaction in the institution from academics with central provision of storage. That being as it may, it was generally agreed that storage was a high priority for Faculty:

*“Yes [storage] is really important. Basically, whenever we have been out to talk to researchers, that's the thing they have latched on to and want to talk about the most.”*

However, the role of the library in delivering storage infrastructure was often said to be limited with a lead being taken by the IT services department.

*“Storage is an important concern for our IT department at the moment as they try and fathom out what their responsibility is going to be in the future and what investment may thus be required.”*

Security was also generally agreed to be an important issue, although often not as pressing as storage. There was an awareness of the need to have provision in place to manage personal or commercially-sensitive data appropriately and the risks of not doing so.

*“Very, very important. Maybe a bit more of a problem on the list of IT guys, “How are we going to include it in our policy?”. We have got a policy, but people still wander about with their memory sticks full of patient data. So yes of course that's a big concern.”*

Awareness of the importance of security was reported to vary significantly across the institution. In some cases, participants themselves did not regard it as a major challenge:

*“It's kind of high on the political agenda just now; however, as a real impact I don't think it's going to take that much work.”*

Once again, however, this particular driver, whilst recognised to be important for the institution, was not generally seen as central for the library except with regard to security of its own systems, including the repository. There was, however, some concern expressed that a number of stakeholders in the institution did not give security of data sufficient priority.

Preservation of data was also regarded as an important issue and a driver for action. However, there was a great deal of uncertainty amongst participants about the practicalities of long-term preservation of research data:

*“Preservation is probably at the level of “It's a good thing, but we don't quite know what we are doing yet”. We certainly don't have much experience of preservation, and but we recognise that it's a good thing, but there are many issues, policy among other things, issues around preservation how long should things be kept for.”*

However, there was an acute awareness amongst many participants of the UK's EPSRC (Engineering and Physical Sciences Research Council) requirement to keep data for 10 years – something which was seen as a major challenge.

As far as the library's involvement in preservation was concerned, the views of many participants agreed with one interviewee who stated that preservation “is an area that the library could and should be involved in”. Mention was made of the professional culture of librarians and its association with preservation and access of content. However, amongst many participants, there was only a hazy understanding of the requirements of digital preservation or of the scale of the problem with regard to data, with more mention of small-scale datasets contained in, for example, spreadsheets than of large-scale scientific data. Nevertheless, a number of participants mentioned the complexity of the digital preservation problem, which many reported they were only beginning to understand in any meaningful way.

It is clear that compliance was also seen as an important issue by all participants. The need to comply with the requirements of UK research funders, particularly EPSRC's requirement for an institutional RDM “roadmap”, had raised awareness of RDM in the institution and driven a significant amount of activity at policy level.

*“Well compliance is really important, yes that's the whole reason we are doing it really. I mean to comply with Research Council guidelines yes. I am not saying the whole reason but that's the main driver, yes.”*

The library's involvement in responding to the EPSRC requirements varied, with the library taking a lead in some cases. However, a number of participants expressed uncertainty about the extent to which compliance would remain an important driver when it was not clear the extent to which it would be monitored by research funders or what the consequences of non-compliance would be.

*“I think we would be slightly relaxed about complying with research funders depending on the consequence of not complying…we are aware that things might change, so we don't want to be too far ahead of the curve, otherwise we could be doing things that turn out not to be required.”*

Whilst the issue of compliance may be recognised as important by the library or the institution, participants reported there may be considerable variation in the way in which it was regarded by academic colleagues.

*“I think the library would recognise it as an important driver but…but in terms of actually having to comply, you will get mixed reactions from researchers.”*

*“That is a very important issue for the university. It is not on the minds of a lot of researchers.”*

However, one important aspect of the design and provision of an advisory service around research data management planning (mentioned by a number of participants as a priority for the library) was the building-in of advice around compliance with funder requirements. This was one aspect of a DMP service which was seen to be an attractive service from an academic point of view.

The driver of research quality was also said to be the subject of a wide variety of views within the institution. One participant pointed out that their institutional RDM policy opened with the statement that high-quality research is underpinned by high-quality research data management, and another commented that much of the input from their Pro-Vice-Chancellor for research had focused on this issue. However, there was some doubt about the extent to which a perspective like this would prompt any changes within institutions:

*“Well you see that is actually I think where there is a certain area of doubt. I am not completely convinced that people perceive this whole research data management sharing agenda as being about improving data quality or research quality, because from their point of view their research is perfectly high quality, thank you very much.”*

Once again, this was not reported to be a priority for many academic staff:

*“It is reasonably important, again it's not necessarily something we are seeing much demand.”*

Although often seen as important for their institutions in principle by participants, research quality enhancement was, therefore, not seen as such an immediate imperative as other drivers. One participant described it as an important idea “whose time had not yet come” since many researchers currently only paid “lip service” to it in principle. Doubt about the extent to which the library could or should be involved in advising on the quality of research was also expressed by participants:

*“Research quality, that's more debatable for us, in terms of what a data service would provide. We provide advice about citations, bibliometrics, impact, that sort of thing, but we are not making judgements about the research quality. That is something that we would leave to the departments, to the research division in terms of putting together research proposals and so on.”*

However, data sharing, particularly in terms of making data openly accessible, was something which most participants were more comfortable advocating. It was clear from their responses that many participants regarded this as amongst the most important of the contributions the library could make to RDM. Both implicit and explicit within the comments of a large number of the participants was the view that the sharing of data was perhaps the key driver for the library's involvement in RDM. Many talked at length about data sharing and the library's general commitment to open access (OA). In fact, there was clearly a strong link made by many participants between RDM and open access, with the two issues (which, of course, do overlap in the area of open data) often being conflated in comments. A number of participants stated that organisationally the same individual or team in the library was responsible for both RDM and OA policy development – a fact which may have promoted the conflation in participants' minds.

Many participants recognised that recent changes in the policy requirements of funders encouraging data sharing gave them greater leverage in raising this issue more widely in their institutions. Some reported support amongst senior managers in the institution. However, although sharing may have been a priority within the library, it was recognised that at an institutional level data sharing was often given a lower priority than other drivers. Regarding its priority, one participant commented:

*“As the library, yes. For researchers, very mixed. I am not sure that there is anyone else in the organisation who thinks about data sharing beyond the library and research [support], [and] some individual researchers.”*

One respondent summarised the variation in views of different stakeholders in their institution in relation to sharing:

*“I think that it fits very well with the library ethos and it would be seen as an important driver, and less so perhaps for…central research support, they would be more interested I think in…compliance rather than the data sharing, and I think researchers are interested in data sharing with…research colleagues, and enlightened researchers are more interested in the wider sharing. I think it's definitely important, but it may not be as an immediately gratifying a driver as something like having really good storage for the researchers.”*

Data sharing was in particular seen to be a controversial issue amongst academics, with significant disciplinary and personal differences highlighted. It was reported that some academics were very negative about data sharing. In many disciplines, it was recognised that there were few incentives for researchers to share their research data particularly at an early stage in research cycle. Sharing was then an issue which was generally not seen as being driven by demand from academics, except in specialised areas. This meant that some participants exercised caution in how they raised the issue with academics.

*“…when I first started talking to people about data management I was slightly wary of making it sound like it was too much about data sharing because I just didn't want to get people's backs up too early because some people's reaction to data sharing is “Oh no I don't want to do that”, and I didn't want that to then turn them off management per se. I mean, let's get the stuff in the repository, let's manage it properly, and then we can worry about whether to make it open or not.”*

Perceptions of the importance of the library's role in activities such as preserving and, particularly, sharing data are at the centre of what might be called the ‘professional jurisdiction’ driver [8], [36]. A theme running through the comments of all participants was the view that RDM was an important agenda and one in which the library should play a major part. Many showed an awareness of activities of library services in other institutions and there was clearly a perception that in professional terms RDM was something the library ‘ought’ to be doing. Regular coverage of RDM issues in professional fora, such as conferences, was clearly in itself acting as a driver for at least some participants to lay claim to an RDM role within their institutions.

Part of this role was focused on creating a ‘story’ around RDM as a coherent concept. RDM is in fact comprised of a number of different strands of activity which might conceivably be seen as separate (albeit related) problems and therefore managed separately. The RDM challenge as being pursued by libraries involves arguing (explicitly or implicitly) for the bundling of these different strands into a single RDM agenda which should then be managed in a coherent way. It is clear that this assumption of the coherence of the RDM agenda has come to inform many of the activities of the participants involved in this research and that of their library organisations and that they see their role partly in terms of advocating such an approach.

## Factors Influencing RDM Developments

As well as describing the different aspects of RDM activity and the key drivers, participants also identified a large number of factors which shape an institutional RDM programme and the library's involvement in it. These might be called ‘Influencing Factors’ since they are intervening conditions which may affect an institutional RDM programme in a variety of complex ways including *either* facilitating *or* constraining action. Key Influencing Factors emerging from this research were:

- Acceptance
- Cultures
- Demand
- Incentives
- Roles
- Governance
- Politics
- Resources
- Projects
- Skills
- Communications
- Context

The first of these, the level of acceptance and prioritisation of RDM as a coherent agenda, is related to the jurisdiction issue above. It was clear from participants' comments that the RDM agenda was not universally accepted in their institutions. Some mentioned scepticism amongst colleagues from other support departments, particularly IT services whose attention was often focused on questions of storage and security only. Others highlighted a lack of engagement from researchers. In either case, it is clear that this lack of acceptance would have a major impact on the way in which an RDM agenda was formed and implemented. In other cases, the RDM agenda may have been recognised but was given lower priority than other key issues. For example, some participants reported difficulties in taking forward the RDM agenda because of the priority given to the research excellence framework (REF) exercise by research support staff and senior academic managers such as pro-vice chancellors.

Added to this, perhaps one of the most significant challenges in implementing institution-wide initiatives was varying cultures and consequent differing working practices. Within any single institution there were seen to be a large number of different cultures across different professions and academic disciplines. Any institution-wide RDM programme, it was recognised, needs to take such varying cultures and practices into account in the way it is designed and implemented. Many of the participants in this study were clearly aware of this issue and mentioned in particular disciplinary differences. Differences manifested themselves in the variety of forms of data being generated and ways in which it was analysed. There were technical differences in areas such as metadata standards and interoperability protocols, but also in cultures around sharing and reuse. However, in many cases, participants were clearly only just beginning to take account of the implications of this for institution-wide RDM programmes. Nevertheless, despite the lack of detailed solutions discussed by participants, the importance of the issues of disciplinary differences should not be underestimated. In many respects, it colours all of the other Influencing Factors.

One of the major challenges implicit in the remarks of many interviewees was that the drivers relating to RDM in many cases operate at an institutional level and are not created by user-level demand in the academic community, regardless of discipline. Apart from data storage, it was clear from responses that demand for RDM-related services amongst users themselves was in fact limited or absent. Instead, participants saw the challenge as lying in *persuading* users to recognise the importance of certain approaches and adopt particular practices associated with RDM, rather than in responding to user demand. A clear understanding of where user demand *does* exist and where there is a perceived *need* for particular services (with or without explicit demand) is certainly therefore an important factor that will shape ongoing RDM activity.

Where there may not be extensive user demand it was seen as essential to identify the key factors that determine behaviours, particularly in terms of incentives. Developing a policy, for example, which includes clear incentives for encouraging desired behaviours, and also clear sanctions to discourage undesired behaviours, was essential. There was clearly uncertainty amongst participants about what such incentives or sanctions should look like at an institutional level in relation to RDM, although the role of the requirements of external funders was seen by many as important in shaping institutional approaches.

Linked to this, a clear understanding of the roles and responsibilities of different stakeholders in institutions emerged as essential in determining the shape and momentum of RDM activities. Just identifying all the stakeholders and their potential roles was problematical. This applied to roles of organisational units, such as the library or IT service, as well as individuals, such as principal investigators or heads of academic departments. Participants reported some uncertainty around all of these in their institutions. There was ongoing uncertainty, for example, in institutions about whether the library was seen as a ‘natural’ place to go for research data management services, although to balance this there was reported to be a high level of acceptance of the library being involved at least in initial stages of RDM programmes around policy development.

Institutional approaches to governance were also recognised to be very important, particularly in terms of decision-making. Universities are often characterised by governance complexity and ambiguity, meaning that decision-making can be a lengthy and opaque process with somewhat indeterminate outcomes. Implementing decisions widely and consistently therefore creates major challenges. Many participants expressed frustration at the delays inherent within University decision-making processes and also of the fact that policy decisions at institutional level did not necessarily result in compliance across the organisation as a whole.

Related to this, the power dynamics within the institution particularly around personal relationships of senior managers were often identified as being important by participants and labelled as “politics”. The relationship between the library director and other senior members of staff, particularly the Pro-Vice-Chancellor for research, Director of research support services and Director of IT services were seen as crucial in taking an RDM programme forward. Securing the attention and time of such individuals and their staff were seen as essential and was a consistent feature of the interviews. As RDM activities matured, relationships between staff in different departments below senior team level became more important and it was here that some participants were beginning to encounter challenges.

Resources available for an RDM programme were seen as crucial. Whilst it was clear that there was still a great deal of uncertainty about the funding and resources required to carry out RDM activities within the institution, there was scepticism about the extent to which new resources would be identified for such activities. Although in some instances business cases for new funding were being developed, most libraries were clearly operating within an environment where efficiency was given a very high priority and gaining additional resources except for fixed-term projects was seen as difficult. Where new staff were required by the library or organisational restructuring was seen as necessary, this would often have to be achieved through repurposing existing resources. Such an atmosphere clearly constrained the scale and scope of RDM activity and services envisaged by many participants.

In many cases, as has been observed, RDM programmes were being focused on fixed-term project activity aiming to deliver specified outputs including policy and guideline documentation and pilot services. These projects were often funded by external agencies, such as Jisc, although a number of participants mentioned internally-funded projects usually also with fixed-term funding. As well as being efficient, these activities were often seen as a vehicle for exploring issues in a manageable way, attempting to tame the scale and complexity of the problem often, for example, with a particular disciplinary focus. It was, however, clear that in the case of many institutions there was still significant uncertainty about next steps following the completion of such projects. In a small number of cases programmes of activity were being developed but were in the early stages. Moving from projects to services, involving scaling up pilots and embedding policies in everyday practices, emerged as a major challenge.

Early activity such as projects often highlighted major skills gaps in organisations, including libraries. Skills and expertise were seen as a major influencing factor on an RDM programme. There was, however, uncertainty amongst participants about the skills required to carry out RDM activities. At this early stage, many libraries had appointed project management staff but were in the process of identifying other areas of expertise necessary to carry out RDM on an ongoing basis. Particular areas identified included advocacy and liaison skills, training skills, as well as technical skills. A large number of participants identified the importance of re-skilling existing staff to be able to operate in the RDM space. However, in some cases, participants reported that they were planning to appoint new staff but this would be limited to only one person or a small number of people (often because of resource constraints). Such constraints were obviously impacting significantly on the shape of the RDM programme being undertaken.

Whether carried out by a new RDM Officer or by other staff within the library (such as subject librarians), communications, and particularly advocacy, were seen as an essential part of any library involvement in RDM. Developing a communication and consultation process in order to determine the content of policies and guidelines and to shape the design of services was seen as essential by participants. The library had an important role in advocacy – articulating the importance of RDM for the institution and its various stakeholders. This could often be achieved by the library its existing channels of communication with particular subject communities established by subject librarians or research-support staff. In addition, it became apparent from interviews that communication within the library about RDM was also important. On occasions, it was clear that the views of senior library staff including the director in relation to research data management may have been out of synchrony with those of other staff. In some cases, following initial negotiation with partners in the University, library directors were devolving RDM activity to their own staff without there being sufficient clarity about what was now expected. Several such staff were participants in the interviews and were clearly going through a process of identifying what next steps were possible and which should be prioritised.

All of these factors affecting the shape of RDM programmes in individual institutions were determined in part by the setting and context within which they were taking place. Research-led institutions were clearly structuring their activities differently from teaching-led institutions, for example. Disciplinary coverage within institutions, varying from multi-disciplinary universities to subject-specific institutes, was also an important factor. It was clear, as one participant put it, that there is a “need to have an understanding of the landscape of your institution” and that institutional setting and context were important influencing factors in the development of an RDM programme.

## Discussion

This analysis reveals a complex picture. While libraries saw that issues around RDM were something to which it was important to respond, their role varied markedly. Any response would be inherently collaborative, but it was clear that solutions developed would vary across different institutions. This is partly because of the existence of a number of different drivers and a large number of inter-related factors influencing how services might be created. It has been suggested that UK institutions have been rather slow to respond to RDM by comparison with the US in particular [9]. The picture from the data in this research reinforces the sense of rather cautious first steps being made, not because of poor leadership (if anything, libraries are leading the way) but constrained by a lack of direction at institutional level. This could have a number of explanations, not least of which is resource limitations. To balance this, however, there was a clear view expressed by participants that the library should be involved in a significant and sustained way in RDM.

**Figure 1:**
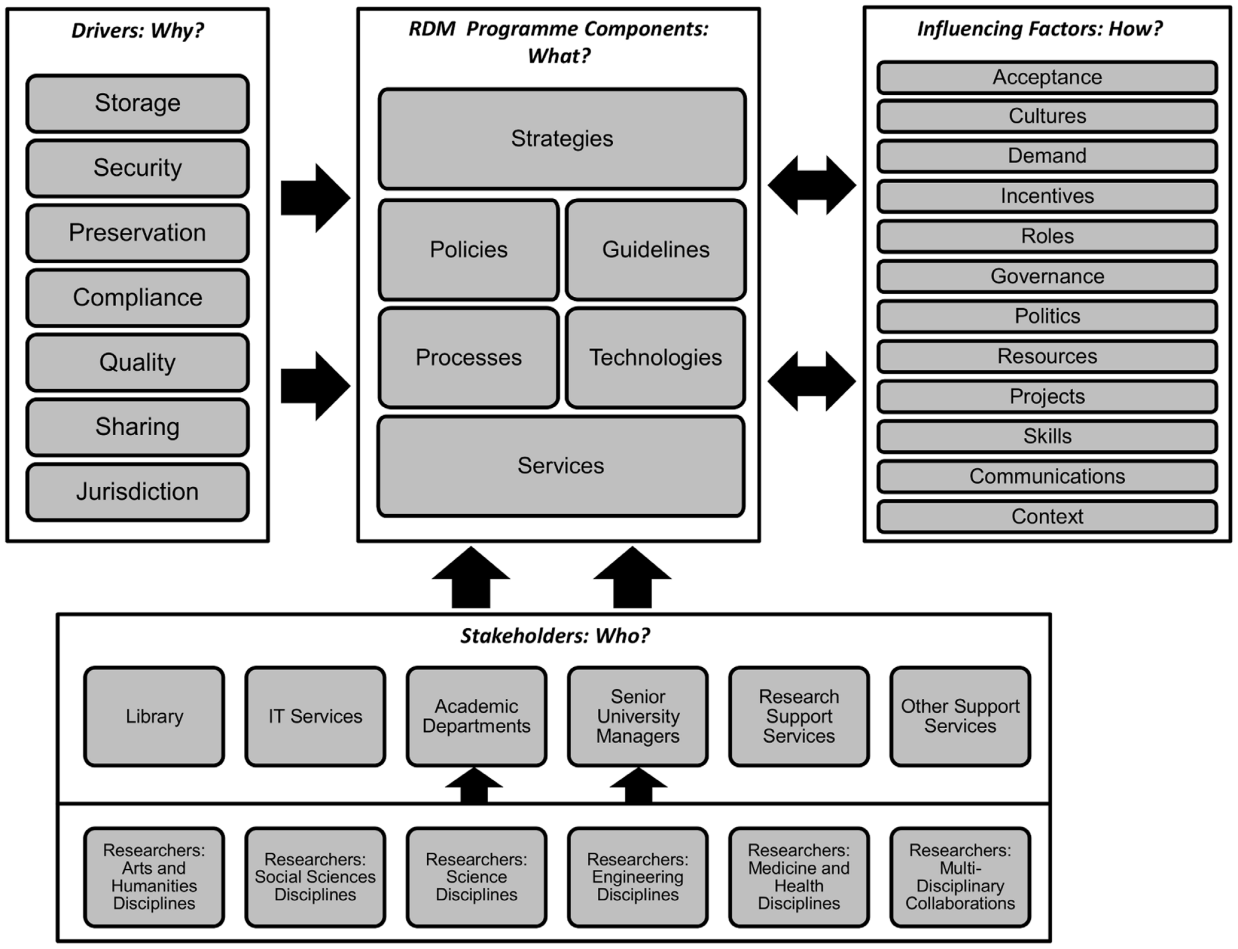
A library-oriented model of institutional RDM.

Combining the findings of this research with issues identified in previous quantitative work [8], a tentative model of an RDM programme within an academic institution has been constructed (Fig. 1). This model helps to clarify the different issues involved in the RDM challenge identifying various layers of activity, multiple stakeholders and drivers, and a large number of factors influencing the implementation of any programme. The model has been created with the library's perspective in mind but also applies more widely across the institution.

The model is intended to address the ‘who?’, ‘what?’, ‘why?’, and ‘how?’ of RDM, particularly in relation to the library's involvement. The ‘Stakeholders’ in the model address the question, ‘Who is involved in institutional RDM?’. They constitute the main actors in the phenomenon being analysed. The ‘RDM Programme Components’ in the model address the question, ‘What does an RDM programme in an institution consist of?’. They constitute the main elements of the phenomenon itself. The ‘Institutional Drivers’ address the question, ‘Why might a programme be carried out?’. They constitute the main causal factors of the phenomenon. The ‘Influencing Factors’ address the question, ‘How will the programme be shaped?’. They constitute the main intervening conditions that affect the phenomenon.

In the model, the different components of an institutional RDM Programme (strategies, policies, guidelines, processes, technologies and services) are shown as logical layers of related activity, from high-level strategic planning to on-the-ground service deployment. Between them, policies and guidelines constitute what might be called a ‘Regulatory’ layer in the model which may often be supported with agreed monitoring mechanisms to measure compliance. Processes and technologies between them constitute a ‘Systems’ layer in the model – systems which are socio-technical in nature.

The different components of an RDM Programme are shaped by a set of Drivers which have been identified by this research: storage, security, preservation, compliance, quality, sharing and jurisdiction. The Drivers identified in the model are not presented in any kind of priority order; notions of priority clearly differ amongst different stakeholders in different institutions and at different times. However, they do move from ‘harder’ technical challenges to ‘softer’ policy and managerial issues. The decision has, however, been made not to distinguish between drivers that are ‘external’ or ‘internal’ to the institution. This study suggests that all of the drivers presented here have external and internal aspects to them with a complex set of issues and influences at play for each driver both within a single institution and beyond.

The RDM programme components and drivers interact with a set of Stakeholders, all of whom have a role in RDM within the institution. Typically, as has been seen, the stakeholders include support staff – the library, IT services, the research support office, other support services (including records management and legal advice functions) – and academic staff – senior university managers (normally led by the Pro-Vice-Chancellor for research, or equivalent) and researchers in academic departments. Amongst academic staff there are a very large number of different researchers with (depending on the institution) different disciplinary approaches, represented in a separate layer in the model. Any institution-wide programme needs to take these disciplinary differences into account. The precise roles of the stakeholders and the relationships between them will, of course, vary between institutions, although it is clear from the research presented here that there are significant commonalities between institutions, including the identity of the players themselves and the particular perspectives they tend to take on RDM.

The implementation of an RDM Programme is affected by a complex set of Influencing Factors which impact on and are impacted by the Programme, either facilitating or constraining action, and influencing its character and direction. They will influence a programme in different ways at different stages (and therefore are not presented in any prioritised order). Each one is in its own right an area of considerable complexity, with additional complexity derived from the interrelationships between different Influencing Factors.

The model aligns well with previous work such as Jones, Pryor and Whyte [25], though operating at a different level. In terms of service components, the current model is at a higher level of generalisation: it does not deal with the specifics of particular types of technologies and services, such as data catalogues, which are more fully developed by Jones et al [25]. In a sense the components set out by their model could be substituted for the “What” part of the model above. At that level it should be clearer how the different professional services' roles might be mapped to the different types of user services, e.g. Active data storage and security – IT; data catalogue – library; Data management planning – Research administration; Guidance, training and support – all. However, the model presented here deals more with the complexity of the underlying drivers and influencing factors that could shape which types of specific service are developed and what they might look like – with a strong acknowledgment that there could be many different outcomes. There are again connections to Whyte's pathway to an RDS [27], although his work focuses on a process of designing a service, as opposed to representing the forces at work which could drive a programme in a particular direction or block easy progress. While it is clearly not the intention of the authors of these previous studies to say “one size fits all”, their approach is to delineate current best practice thinking. The current model makes it clearer why in practice very different patterns of support service might emerge, or indeed none at all, because it retains a complex sense of the underlying drivers and acknowledges the constraints on putting services in place.

In any particular RDM institutional programme it is reasonable to assume that successful outcomes will be achieved if all of the central components identified (strategies, policies, etc) are implemented by the appropriate stakeholders, in line with the drivers, and taking into account the relevant influencing factors. However, there is no single development pathway. Many institutions are evidently formulating and documenting policies as an initial stage and following this up with the creation of more detailed guidelines as processes and technologies begin to be put in place. There is activity in the areas of technology development and piloting of services. At the same time, work to identify and allocate required resources, clarify roles and responsibilities, and enhance necessary skills development are under way. Commonly, this is being done through project activity, which is also enabling some advocacy to take place, but it is clear that the scaling and operationalisation of activity remains challenging. Moreover, ensuring that all the different components of the programme (policies, guidelines, processes etc) are all developing at a consistent rate and with appropriate levels of compatibility and integration is also challenging. Managing the different strands of activity being carried out by different actors (the library, IT services, academic departments etc) together to form a coherent institutional approach without unnecessary duplication or incompatibility is becoming a concern for many.

This model serves to encapsulate reported RDM activity within institutions, and it may also act as a diagnostic tool. If an RDM programme is stalled, for example, it may help to analyse progress against the model in order to identify areas where little or no activity has been focused or where there is a significant constraining influence. Approaches can then be developed to address these issues.

Of course, discussion on the questions, ‘who?’, ‘what?’, ‘why?’, and ‘how?’ begs the question, ‘when?’. This is not been built into the model since the participants in the research presented in this study were very uncertain about timescales. Whilst quantitative data reported by Cox and Pinfield indicates that libraries expect to have made significant progress in RDM “in the next three years” [8], this may often be a statement of aspiration rather a summary of specific plans. The fact that it is “early days” and that, apart from policy development, libraries in particular and institutions in general are still “feeling their way” with RDM means that ‘when?’ is still very much an open question.

One under-developed aspect of the model is the treatment of the way that any initiative needs to be adaptive to the diverse disciplinary cultures of research communities [27]. For example, it is well understood that some disciplines already have a deeply ingrained culture of sharing data openly (physics) or reuse of secondary data (economics); others have well-developed data management practices, driven by the use of personal data (health sciences); still others, such as in some of the arts and humanities, rarely even use the term “data”. Such issues are incorporated in but are not central to the current model. This is partly a function of the data used to produce it: namely, librarians in the early days of planning RDM activities, before intense engagement with research communities around initial services. Although participants were conscious of the importance of disciplinary differences, the ways in which these should be reflected in the detail of institutional services was still often unclear. Data based on the perspective of other stakeholders, such as researchers, but also other professional services, might also lead to the model being refashioned. As has been stressed, the data reported here are the library perspective on the issues: this viewpoint is important in itself, but it is a partial perspective. So the model should be seen as a first tentative formulation based on one dataset – but one that does accurately capture the perspective of libraries on the early days of developing RDM.

At the same time, it seems plausible that elements of the model may be generalised to apply to other institutional information-related initiatives in higher education organisations. Whilst the specific drivers and stakeholders will differ, the programme components and influencing factors are likely to apply in other cases, although levels of complexity may differ depending on the specific initiative. Initial analysis suggests that it would, for example, be applicable to the development of an institutional open access programme in this form. Here the development of a strategic approach to open access accompanied by appropriate policies and guidelines, development of relevant technologies and processes, and provision of supporting services might be seen to be influenced by similar factors to RDM. Furthermore, other institutional initiatives outside of the information field also arguably follow a similar pattern. Testing the utility of this model for other developments in HE, both information-related and other initiatives, may be a useful area of further research.

## Conclusion

Research data management is a complex issue involving multiple activities carried out by various actors addressing a range of drivers and influenced by a large set of factors. The analysis and modelling of developments presented in this study contribute to the understanding of the ways in which institutions are addressing the problem by presenting in detail the perspective of one major stakeholder group. Whilst the analysis has focused on the activities of libraries in particular, it illustrates more generally how different actors are adapting their roles to participate in emergent RDM programmes. However, major uncertainties remain in how the various stakeholders relate to each other, where strategic priorities lie, and how socio-technical systems should best be designed to deliver value to the organisation in particular and research community in general. Library activity, currently concentrated in areas such as advocacy and policy development, and moving into new areas including the support functions and creation of new systems, still has an important element of provisionality about it. As RDM matures in universities, further quantitative and qualitative work will be needed to understand the shape of activities and the roles of different actors in order to inform ongoing development.

## References

[1] McAfee A, Brynjolfsson E (2012) Big data: The management revolution. Harv Bus Rev 90:60–68. 23074865

[2] Laney D (2001) 3D data management: Controlling data volume, velocity and variety. Stamford, CT: META Group. Available: http://blogs.gartner.com/doug-laney/files/2012/01/ad949-3D-Data-Management-Controlling-Data-Volume-Velocity-and-Variety.pdf.

[3] Borgman CL (2012) The conundrum of sharing research data. J Am Soc Inf Sci Technol 63:1059–1078 Available: http://doi.wiley.com/10.1002/asi.22634 Accessed 20 February 2014.

[4] Royal Society (2012) Science as an open enterprise: Final report. Available: https://royalsociety.org/policy/projects/science-public-enterprise/Report/. Accessed 2014 May 1.

[5] EPSRC (2011) EPSRC policy framework on research data. Engineering and Physical Science Research Council. Available: http://www.epsrc.ac.uk/about/standards/researchdata/. Accessed 2014 May 1.

[6] NIH (2003) Final NIH statement on sharing research data. National Institutes of Health. Available: http://grants.nih.gov/grants/guide/notice-files/NOT-OD-03-032.html. Accessed 2014 May 1.

[7] Lewis MJ (2010) Libraries and the management of research data. In: McKnight Seditor. Envisioning future academic library services. London: Facet. Available: http://eprints.whiterose.ac.uk/11171/1/LEWIS_Chapter_v10.pdf. Accessed 2014 Jan 9.

[8] Cox AM, Pinfield S (2014) Research data management and libraries: Current activities and future priorities. J Librariansh Inf Sci. Available: http://lis.sagepub.com/cgi/doi/10.1177/0961000613492542. Accessed 2014 Mar 30.

[9] Corrall S, Kennan M, Afzal W (2013) Bibliometrics and research data management services: Emerging trends in library support for research. Libr Trends 61:636–674 Available: http://muse.jhu.edu/journals/library_trends/v061/61.3.corrall02.html Accessed 2013 Dec 23.

[10] Tenopir C, Birch B, Allard S (2012) Academic libraries and research data services: Current practices and plans for the future: An ACRL white paper. Chicago, IL: Association of College and Research Libraries. Available: http://www.ala.org/acrl/sites/ala.org.acrl/files/content/publications/whitepapers/Tenopir_Birch_Allard.pdf.

[11] Pryor G, Jones S, Whyte A, editors (2013) Delivering research data management services: Fundamentals of good practice. London: Facet.

[12] Pryor G, editor (2012) Managing research data. London: Facet.

[13] Auckland M (2012) Re-skilling for research: An investigation into the role and skills of subject and liaison librarians required to effectively support the evolving information needs of researchers. London: Research Libraries UK. Available: http://www.rluk.ac.uk/wp-content/uploads/2014/02/RLUK-Re-skilling.pdf.

[14] Nielsen HJ, Hjørland B (2014) Curating research data: The potential roles of libraries and information professionals. J Doc 70:221–240 Available: http://www.emeraldinsight.com/journals.htm?issn=0022-0418&volume=70&issue=2&articleid=17105220&show=html Accessed 2014 May 2.

[15] Whyte A, Tedds J (2011) Making the case for research data management. Edinburgh: Digital Curation Centre. Available: http://www.dcc.ac.uk/webfm_send/487.

[16] Delserone LM (2008) At the watershed: Preparing for research data management and stewardship at the University of Minnesota libraries. Libr Trends 57:202–210 Available: http://muse.jhu.edu/journals/library_trends/v057/57.2.delserone.html Accessed 2014 May 1.

[17] Henty M (2008) Dreaming of data: The library's role in supporting e-research and data management. Australian Library and Information Association Biennial Conference, Alice Springs. Available: http://apsr.anu.edu.au/presentations/henty_alia_08.pdf.

[18] Corrall S (2012) Roles and responsibilities: Libraries, librarians and data. In: Pryor Geditor. Managing research data. London: Facet. pp.105–133.

[19] Cox AM, Verbaan E, Sen BA (2013) Upskilling liaison librarians for research data management. Ariadne 70 Available: http://www.ariadne.ac.uk/issue70/cox-et-al.

[20] Lyon L (2012) The informatics transform: Re-engineering libraries for the data decade. Int J Digit Curation 7:126–138 Available: http://ijdc.net/index.php/ijdc/article/view/210 Accessed 2013 Nov 7.

[21] Procter R, Halfpenny P, Voss A (2012) Research data management: Opportunities and challenges for HEIs. In: Pryor Geditor. Managing research data. London: Facet. pp.135–150.

[22] Tenopir C, Sandusky RJ, Allard S, Birch B (2013) Academic librarians and research data services: Preparation and attitudes. IFLA J 39:70–78 Available: http://ifl.sagepub.com/content/39/1/70.short Accessed 2014 May 1.

[23] Tenopir C, Sandusky RJ, Allard S, Birch B (2014) Research data management services in academic research libraries and perceptions of librarians. Libr Inf Sci Res 36:84–90 Available: http://www.sciencedirect.com/science/article/pii/S0740818814000255 Accessed 2014 Aug 15.

[24] Cox AM, Pinfield S, Smith J (2014) Moving a brick building: UK libraries coping with research data management as a “wicked” problem. J Librariansh Inf Sci. Available: http://lis.sagepub.com/content/early/2014/05/13/0961000614533717.abstract. Accessed 2014 May 21.

[25] Jones S, Pryor G, Whyte A (2013) How to develop RDM services: A guide for HEIs. Edinburgh: Digital Curation Centre.

[26] Jones S (2014) The range and components of RDM infrastructure and services. In: Pryor G, Jones S, Whyte Aeditors. Delivering research data management services. London. pp.89–114.

[27] Whyte A (2014) A pathway to sustainable research data services: From scoping to sustainability. In: Pryor G, Jones S, Whyte Aeditors. Delivering research data management services. London: Facet. pp.59–88.

[28] Mayernik M, Choudhury G, DiLauro T, Metsger E, Pralle B, et al (2012) The data conservancy instance: Infrastructure and organizational services for research data curation. D-Lib Mag 18 Available: http://www.dlib.org/dlib/september12/mayernik/09mayernik.html Accessed 2014 Apr 26.

[29] Rice R, Haywood J (2011) Research data management initiatives at University of Edinburgh. Int J Digit Curation 6:232–244 Available: http://www.ijdc.net/index.php/ijdc/article/view/194 Accessed 2014 Apr 26.

[30] Wilson JAJ, Martinez-Uribe L, Fraser MA, Jeffreys P (2011) An institutional approach to developing research data management infrastructure. Int J Digit Curation 6:274–287 Available: http://ijdc.net/index.php/ijdc/article/view/198 Accessed 2014 Apr 26.

[31] Carlson J, Garritano J (2010) E-science, Cyberinfrastructure and the Changing Face of Scholarship: Organizing for New Models of Research Support at the Purdue University Libraries. In: Walter S, Williams Keditors. The Expert Library: Staffing, Sustaining, and Advancing the Academic Library in the 21st Century. Chicago, IL: Association of College and Research Libraries. pp.234–269. Available: http://docs.lib.purdue.edu/lib_research/137. Accessed 2014 May 1.

[32] Shen Y, Varvel VE (2013) Developing data management services at the Johns Hopkins University. J Acad Librariansh 39:552–557 Available: http://www.sciencedirect.com/science/article/pii/S0099133313000815 Accessed 2014 Apr 30.

[33] Treloar A, Choudhury GS, Michener W (2012) Contrasting national research data strategies: Australia and the USA. In: Pryor Geditor. Managing research data. London: Facet. pp.173–204.

[34] Braun V, Clarke V (2006) Using thematic analysis in psychology. Qual Res Psychol 3:77–101 Available: http://www.tandfonline.com/doi/abs/10.1191/1478088706qp063oa Accessed 2013 Nov 7.

[35] Kennan MA (2011) Learning to share: Mandates and open access. Libr Manag 32:302–318 Available: http://www.emeraldinsight.com/journals.htm?issn=0143-5124&volume=32&issue=4/5&articleid=1926028&show=html Accessed 2013 Aug 5.

[36] Abbott A (1988) The system of professions. Chicago, IL: University of Chicago Press.

